# The Biomarkers Changes in Serum and the Correlation with Quantitative MRI Markers by Histopathologic Evaluation of the Cartilage in Surgically-Induced Osteoarthritis Rabbit Model

**DOI:** 10.1371/journal.pone.0124717

**Published:** 2015-04-17

**Authors:** Houdong Zuo, Lingxia Jiang, Nan Qu, Jianhua Wang, Xiaojiang Cui, Weiwu Yao

**Affiliations:** 1 Department of Radiology, Shanghai Jiao Tong University Affiliated Sixth People’s Hospital, Shanghai, China; 2 Department of Radiology, Tenth People's Hospital of Tongji University, Shanghai, China; 3 Department of Orthopedics, Xin Hua Hospital Affiliated to Shanghai Jiao Tong University School of Medicine, Shanghai, China; 4 Department of Surgery, Samuel Oschin Comprehensive Cancer Institute, Cedars-Sinai Medical Center, Los Angeles, California, United States of America; University of Massachusetts Medical, UNITED STATES

## Abstract

**Purpose:**

To investigate the biomarkers change in serum and the correlation with quantitative MRI markers by histopathologic evaluation of the cartilage in surgically-induced osteoarthritis(OA) rabbit model.

**Materials and Methods:**

Thirty-six mature New Zealand rabbits were used. Eighteen rabbits were divided into six groups randomly and equally and subjected to surgery using the improved Hulth method. The other eighteen rabbits were also allocated into six groups randomly and equally which served as the control. At multiple time points after surgery, the BMP-2, CTX-II and COMP levels in the serum were analyzed by ELISA, and quantitative MRI was performed. Histopathology was examined with HE, and Mankin scores were assessed. The changes in the biochemical biomarkers and imaging markers in the OA groups were compared with those in the control groups using paired-samples T tests. The correlation of quantitative MRI markers with biomarkers and Mankin scores were analyzed. The analysis of Mankin scores was conducted with non-parametric wilcoxon signed rank tests.

**Results:**

The BMP-2 levels were increased at various times after surgery, and significant differences were observed between the OA and control groups(all the *P* values <0.001). CTX-II levels were significantly elevated at several intervals after surgery, including W2, W8, W12, W16 and W20(*P*=0.019, 0.004, 0.007, <0.001 and 0.016 respectively), but not at W4(*P*=0.764). Significant differences in the COMP levels from W2 to W20 were observed between the OA and the control groups(*P*<0.001, <0.001, <0.001, <0.001,=0.002 and =0.004 respectively). The T2 values increased at W8 post-surgery and were significantly different between the OA and control groups(*P*=0.001, <0.001, <0.001 and <0.001 respectively). T2* values increased from W2 to W20 and were significantly different between the control and OA groups(*P*=0.002, =0.001, <0.001, <0.001, =0.001 and <0.001 respectively). T2 values had significant correlation with BMP-2 and CTX-II(*P*<0.001 and =0.014), except COMP(*P*=0.305)., while the correlation of T2* values with BMP-2, CTX-II and COMP was significant(*P*=0.043, 0.005 and 0.025 respectively). In addition, a positive correlation of T2 values and Mankin scores was observed(*P*<0.001).

**Conclusion:**

With the relevance of the multiple time point analysis of the serum biomarkers and imaging markers compared with histological findings, BMP-2, CTX-II and COMP combined with T2 and T2* can be used to reflect and monitor OA progression potentially.

## Introduction

Knee OA has become a very common musculoskeletal disease that affects the health and quality of life of millions of people [1]. At present, methods to evaluate OA such as the WOMAC score and the Knee Society score can provide useful information about the current condition of knee OA and facilitate the adoption of optimal treatment methods [2]. However, these scores are insufficient for evaluating disease activity and progress or for predicting the prognosis of knee OA.

With the development of molecular biology, OA biomarkers have attracted increasing attention as objective indices for early diagnosis of knee OA and prediction of the degree of progression and the prognosis. Currently, many types of joint biomarkers, including bone morphogenetic protein 2(BMP-2)[3–4], C-telopeptide of type II collagen(CTX-II)[5–7] and cartilage oligomeric matrix protein(COMP)[8–9], have been identified and thoroughly studied. These biomarkers have great potential in both medicine and medical economics.

BMP-2 is a low molecular weight glycoprotein and classified as a morphogen and belongs to the expanding TGF-β protein superfamily which plays a key role in the pathogenesis of cartilage [10–11], protection of cartilage against destruction and formation of new cartilage. BMP-2 has pleiotropic functions that range from extraskeletal and skeletal organogenesis to bone generation and regeneration [12–13].

CTX-II is the degradation product which is produced in OA with the the destruction of type II collagen in cartilage and finally excreted in the urine. It become one of the most studied markers currently. CTX-II content is higher in the urine of patients with hip OA than in healthy subjects and is also higher in rapidly developing OA cases than in slowly developing cases [14]. Moreover, CTX-II is associated with both the prevalence and the progression of radiographic OA of the knee and hip [15–16]. Another serum factor of the cartilage, COMP has the potential to be a prognostic marker of disease progression. High COMP levels that persisted over a 3-year study period in patients with radiographic progression of OA indicated differences in disease activity that were detectable throughout the entire follow-up interval [8–9, 17].

Nowadays, MRI renders non-invasive observation and evaluation of the cartilage. Some techniques are used in the evaluation of the cartilage, such as fat suppressed three-dimensional spoil gradient-recalled sequence(3D-SPGR), T2 mapping, T2* mapping, and so on. 3D-SPGR is useful for the cartilage, especially in detecting the defect of the cartilage. However, the usage of 3D-SPGR mainly focus on the morphology and can’t realize the quantitative assessment of the cartilage really [18–19]. The technique of T2 mapping and T2* mapping can evaluate the cartilage quantitatively. Nowadays, quantitative MRI markers including T2 and T2* values are hot focus which can reveal the biochemical components changes of cartilage undergoing early degeneration [20–21].

The aim of this study was to investigate the biomarkers changes in serum and the correlation with quantitative MRI markers by histopathologic evaluation of the cartilage in the surgically-induced osteoarthritis rabbit model. The evolution of three biomarkers concentrations and quantitative MRI markers was followed and observed in a 20-week longitudinal study with the rabbit OA model induced by the improved Hulth method [22]. We anticipated that our findings could provide useful information for the clinical management and follow-up of OA and lay preliminary foundation of noninvasive observation of OA in the future.

## Materials and Methods

The experiments were performed in accordance with relevant guidelines and regulations. All rabbits were conducted in compliance with the “Guide for the Care and Use of Laboratory Animals” published by the National Institutes of Health (NIH Publication No. 85–23, revised 1985). This study was approved by the Animal Care And Use Committee of Shanghai Jiao Tong University Affiliated Sixth People’s Hospital (License No. SYXK 2011–0128) and conducted in compliance with the regulations of the current version of the China Law on The Protection of Animals. Thirty-six New Zealand White (NZW) male rabbits (24 weeks old) weighing 3.0±0.5 kg were used in this study. The differences in the stages of the estrous cycle, the stages of such as development pregnancy and lactation, and interactions with male rabbits among female rabbits may affect the results. Therefore, in order to minimize the effect of the gender difference on the results, the male rabbits were used. A previous study on the disease also used a male rabbit model [5]. Eighteen rabbits served as control animals (n = 3 for each group), and the others were divided into six experimental OA groups(n = 3 for each group) randomly and equally corresponding to different time intervals after surgery (W2, W4, W8, W12, W16 and W20). BMP-2, CTX-II and COMP levels were tested, and MRI was performed at different time intervals. After that, they were sacrificed at the same time for each group and the cartilage was removed and subjected to histopathologic examination.

### Experimental osteoarthritis model

The improved Hulth method of experimental OA was used. The key procedures of the Hulth method involve a medial parapatellar incision, followed by opening knee joints, transecting anterior and posterior cruciate ligaments and excising medial meniscus [22]. The improved Hulth method does not have the posterior cruciate ligament transection. The surgery was performed on the left knees [5]. The surgical procedure for the experimental groups was performed under sterile conditions. Pentobarbital methanol solution (2.5% pentobarbital methanol solution with normal saline, 1:1) was used for anesthesia. All the rabbits of the OA groups were injected through the ear vein with 1.0 ml/kg of anesthetic. The anesthesia was considered successful when the muscular tension and corneal reflex disappeared. An anteromedial skin incision of approximately 3–4 cm in length was then made over the left knee. The medial meniscus and medial collateral ligament were resected from inside the ligamentum patellae. Cares were taken to protect and retract the vascular intraarticular fat pad. Then, the anterior cruciate ligament was transected (ACLT). The medial lateral stress experiment and the anterior drawer test were performed. The surgery was considered successful when a positive sign was presented. The joint was washed with sterile saline solution. The capsule and the synovium were then closed together with 4.0 interrupted Vicryl, and the skin was closed step by step. The rabbits were postoperatively administered 800000 units penicillin (1 time/day for 7 days) via intramuscular injection and kept in single-subject cages. Analgesics were administered as needed. The rabbits were allowed to bear weight as tolerated. With control groups, sham surgery was operated. We just open the cavity of the joint, but no disturbance was performed with the cartilage. After the surgery, in order to remain the same level of behavior and activity, all the rabbits were received the same forced movement or activity (two times per day,20min per one time) until the end of the study(20 consecutive weeks).

### Testing of biochemical markers

1. Serum collection and preservation. Pre- and post-surgery, 3.0 ml blood was drawn from the central artery of the rabbit ear. Afterwards, the blood was collected in test tubes, labeled, centrifuged (4000 r/min, 5 min) and finally preserved at -20°C.
2. Serum factors test. An enzyme-linked immuno sorbent assay (ELISA) was used to test the concentrations of BMP-2, CTX-II, and COMP in the serum. ELISA kit was purchased from Wuhan EIAab Science Co.,Ltd (sensitivity: ≥10 pg/ml). The microtiter plate provided in this kit has been pre-coated with an antibody specific to BMP-2, CTX-II and COMP. Samples are then added to the appropriate microtiter plate wells with a biotin-conjugated polyclonal antibody preparation specific for biomarkers and Avidin conjugated to Horseradish Peroxidase (HRP) is added to each microplate well and incubated. Then a TMB substrate solution is added to each well. Only those wells that contain those biomarkers, biotin-conjugated antibody and enzyme-conjugated Avidin will exhibit a change in color. The enzyme-substrate reaction is terminated by the addition of a sulphuric acid solution and the color change is measured spectrophotometrically at a wavelength of 450 nm. The concentration of CTX-II in the samples is then determined by comparing the O.D. of the samples to the standard curve.

### MRI protocol and image processing

**Fig 1:**
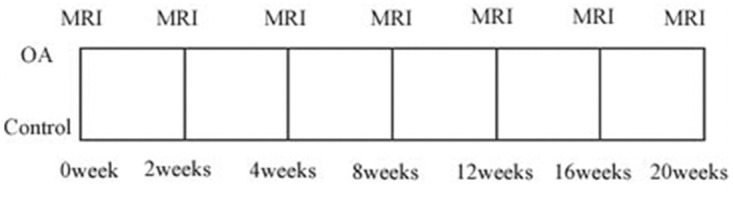
The overview chart of the design in this study. MRI scanning was evaluated at different time points and histologic analysis was assessed at the same time (black arrows). ACLT = Anterior cruciate ligament transection. Control = Control group. n = 18 for ACLT and control group.

The experimental protocol is illustrated in Fig 1. All the knee joints were imaged on a Siemens Verio 3.0T MR scanner with a special animal knee joint coil (CG-RBC18-H300-AS). Pd-TSE, 4 echo SE (T2 mapping) and 5 echo GRE (T2* mapping) sequences were used. The following parameters were used: TR 3300 ms, TE 22 ms, average 2, and scanning measuring time (TA) 5.41 min for Pd-TSE; TR 1000 ms, TE 11.5, 23, 34.5, and 46 ms, average 2, and TA 6.52 min for T2 mapping; and TR 445 ms, TE 4.36, 11.9, 19.44, 26.98, and 34.52 ms, average 2, and TA 5.43 min for T2* mapping. The following geometric parameters were the same for all the sequences: FOV, 140×140 mm; matrix, 384×384; phase resolution, 100%; slice thickness, 2.5 mm and slices, 12.

Before MR scanning, pentobarbital methanol solution (1.0ml/kg) was administrated by ear vein injection slowly. After anaesthetized, the rabbits were fixed in the supine position on the bed. Then both knees were put in the center of the coil and fixed with medical plasters. After scanning, all the MR images were sent to an independent workstation and the analysis was performed on a workstation(ADW4.0). The imaging analysis was performed by two well-trained radiologists. The middle and more clear slices were selected in the lateral tibiofemoral joint for measurement in Pd-TSE. Ten regions of interest (ROIs) were selected according to WORMS including the medial femur (middle and posterior), lateral of the femur (middle and posterior), the medial tibia (anterior, middle and posterior), and lateral of the tibia (anterior, middle and posterior)[23].

To reduce the effect of non-cartilage components or the the partial volume effect, the borders of the articular cartilage and menisci were not drawn in the ROIs. The precise matching of pixel-to-pixel in orientation and measurement of the ROIs were processed on the PACS workstation. The mean values of the signal intensity in the ROIs were measured. ROIs were drawn manually on each region. Three times measurements were performed in each ROIs and the mean values were calculated. All the T2 and T2* values were measured by two well-trained radiologists(HDZ, LXJ with 10 and 18 years of experience) separately and repeatedly(WWY, JHW with 20 and 15 years of experience) in a blinded fashion.

Due to the advantage of reducing fitting error in low signal-to-noise MR images with regional-based method [24], The T2 and T2* relaxation times of the cartilage were calculated zone by zone in the selected ROIs using the least-square, single-exponential curve-fitting method on a MATLAB 8.0 (MathWorks, Natick, MA, USA) software platform. All the data shown in this manuscript are the mean values.

### Histopathology preparation

Samples of articular cartilage were removed with a scalpel from the central portion of weight-bearing areas of femurs subjected to improved Hulth method operations and non-operated control femurs. General observation revealed that joint fluid, synovial swelling and regions of cartilage damage varied among the rabbits. Samples were imbedded in 10% formaldehyde solution for fixation. Then, the samples were decalcified until they were softened. Cares were taken to harvest specimens from consistent locations. However, in some severe cases, full-thickness involvement of the articular cartilage was found, and the samples were obtained from adjacent regions instead. Serial 1 mm sections were cut from the samples and then imbedded in petroleum wax and stained with HE. Observations were made using a light microscope to evaluate the cartilage structure, and Mankin scores of articular cartilage under 3 different views (20×) were assigned with reference to the Mankin pathologic score criterion [25]. The overview design of MRI and histologic analysis was seen in Fig 1.

### Statistical analysis

The continuous data were expressed as the mean±standard deviation (SD). Paired-samples T tests were used to analyze the changes of BMP-2, CTX-II, COMP, T2 and T2* values compared with the control groups(2-detailed,95% CI). The normality was tested by Shapiro-wilk, p value >0.1 was considered the data was normally distributed. Statistical analysis of Mankin scores across these different times groups and pairwise differences were conducted using a wilcoxon signed rank tests. The linear regression analysis was performed to evaluate the correlation of quantitative MRI markers with biomarkers and Mankin scores with software SPSS16.0 package for Windows (SPSS Institute, Chicago, IL, USA). All tests for statistical significance were 2-tailed with an α level of 0.05 and a 95% confidence interval. The level of the agreement was determined by calculating the kappa coefficients with MedCalc (Version 14.12.0),and kappa values of >0.75, 0.4–0.75, and <0.40 were designated as representing excellent, moderate, and poor reliability, respectively. The root-mean-square average coefficient of variation (RMSA-CV) was used to evaluate the reproducibility of the T2 and T2* measurements, RMSA-CV was determined by √((∑CV^2^)/n) where intra-subject CV was calculated by dividing the SD of T2 and T2* values by the mean of the subjects’T2 and T2* values from for each ROI, where n was the number of subjects. RMSA-CV values less than 10% were considered as good.

## Results

### Biochemical marker changes

**Fig 2:**
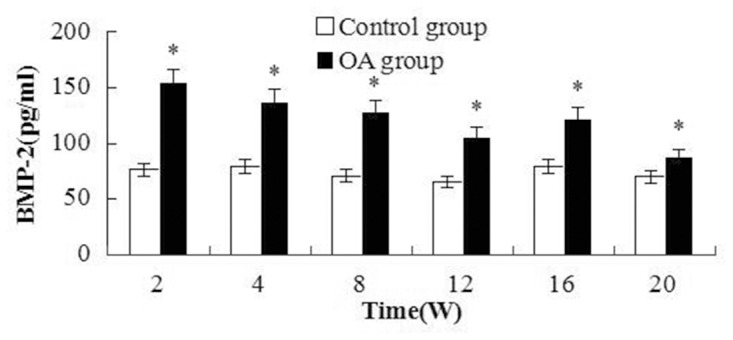
Plot of the changes in the BMP-2 levels in serum at different post-surgery time points. The BMP-2 levels peaked at times W2 and W16. Significant differences were found between the OA and control groups. Asterisks indicate significant differences with paired-samples T tests.

**Fig 3:**
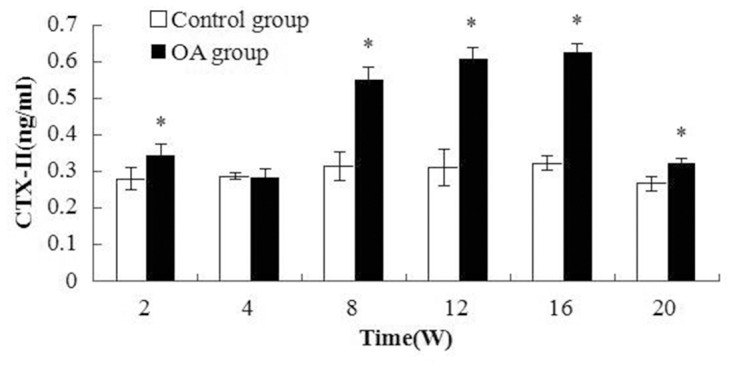
Plot of CTX-II level changes in the serum. The CTX-II levels for the OA groups were higher than that for the control group, except at time point W4. The CTX-II level peaked at W16. Significant differences were found between the control group and all the OA groups except the group evaluated 4 weeks post-surgery. Asterisks indicate significant differences with paired-samples T tests.

**Fig 4:**
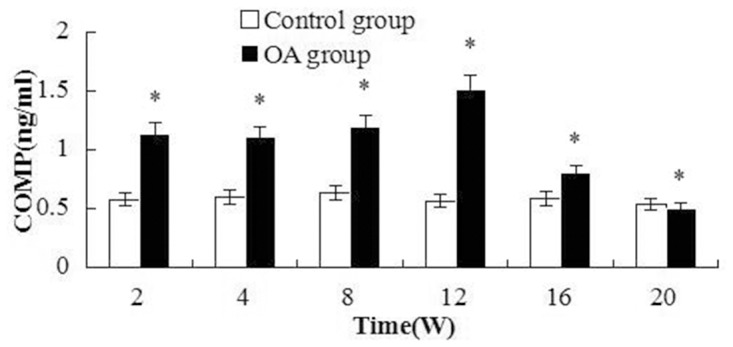
Changes in COMP levels in the serum at different points after surgery. The COMP level peaked at time point of W12 and decreased at W16. Significant differences were found between the control group and all OA groups except the W20 group. Asterisks indicate significant differences with paired-samples T tests.

All the markers level changes pre- and post-surgery were illustrated in Fig 2, Fig 3 and Fig 4. Two peaks of BMP-2 level appeared in Fig 2, the first peak appeared at W2, then the level decreased, but it was higher than that the control. W16 was another peak. And significant differences were found between the OA and control group(all the *P* values <0.001). As to CTX-II, the first peak appeared during the first two weeks, and the second peak arose between 8 to 16 weeks after surgery, the highest level was at W16. The significant differences were observed between the OA and control at multiple time intervals except W4(*P* = 0.019, 0.004, 0.007, <0.001, 0.016 and 0.764 respectively). Regarding COMP, the level was elevated from W2 to W20 after surgery, and the significant differences were found compared with the control groups(*P*<0.001, <0.001, <0.001, <0.001, = 0.002 and = 0.004 respectively). After W20, the level was close to the level of the control group.

#### Quantitative MRI evaluation

**Fig 5:**
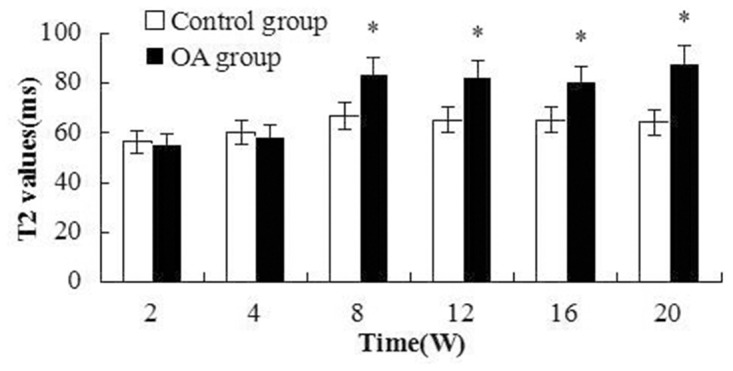
T2 values were elevated after W8, which suggests that the increase in T2 values is related to the severity of OA. Asterisks indicate significant differences with paired-samples T tests.

**Fig 6:**
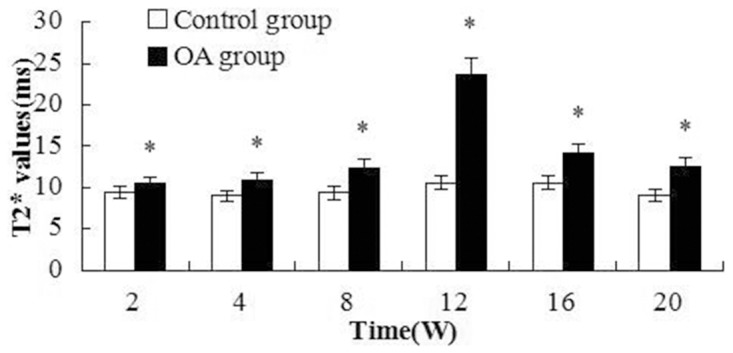
T2* values were elevated after W2 compared with the control group, but significant differences appeared from W8 to W20. Asterisks indicate significant differences with paired-samples T tests.

**Fig 7:**
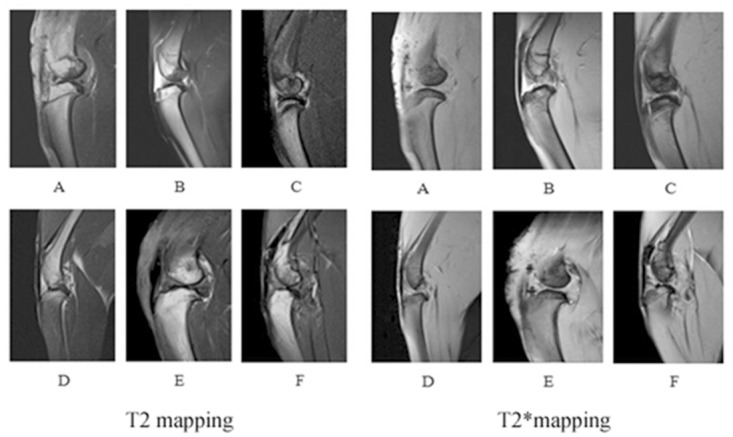
T2 mapping images (A-F) and T2* mapping (A-F) images of rabbit cartilage at different time points after surgery (W2, W4, W8, W12, W16, and W20). The cartilage was integral and had moderate to slightly high linear homogeneous signal (A-B). The cartilage had moderate to slightly high signal and poor contrast with adjacent synovial fluid, adipose tissue and myeloid tissue(C-D). The cartilage was not integral and had slightly high heterogeneous signal(E-F).

The mean T2 and T2 * relaxation time values of the control and OA groups were demonstrated and elucidated in Fig 5 and Fig 6. The changes of T2 and T2* values were not remarkable. The T2 values increased at W8 post-surgery and were significantly different between the OA and control groups(*P* = 0.001, <0.001, <0.001 and <0.001 respectively). T2* values increased from W2 to W20 and were significantly different between the control and OA groups (= 0.002, = 0.001, <0.001, <0.001, = 0.001 and <0.001 respectively). The first peak of T2 values appeared at W8 after surgery, whereas the T2* value peak appeared at W12. T2 and T2* mapping images of the rabbit cartilage are shown in Fig 7.

The kappa values of the inter-group were 0.919(95% CI:0.895–0.943) and 0.735(95% CI:0.669–0.801) for T2 and T2* measurements respectively, indicating the moderate to excellent agreement and reliability between the two groups. The RMSA-CV of the T2 measurements in the control and OA were 3.43% and 2.72% respectively, while for T2*, the RMSA-CV were 7.75% and 7.71%. The values suggested good reproducibility.

### Histopathological analysis and Mankin scores

#### Microscopic observation

The cartilage cells of the control were distributed uniformly, and the tidemark was integral. The matrix was dyed evenly without loss of dye.

**Fig 8:**
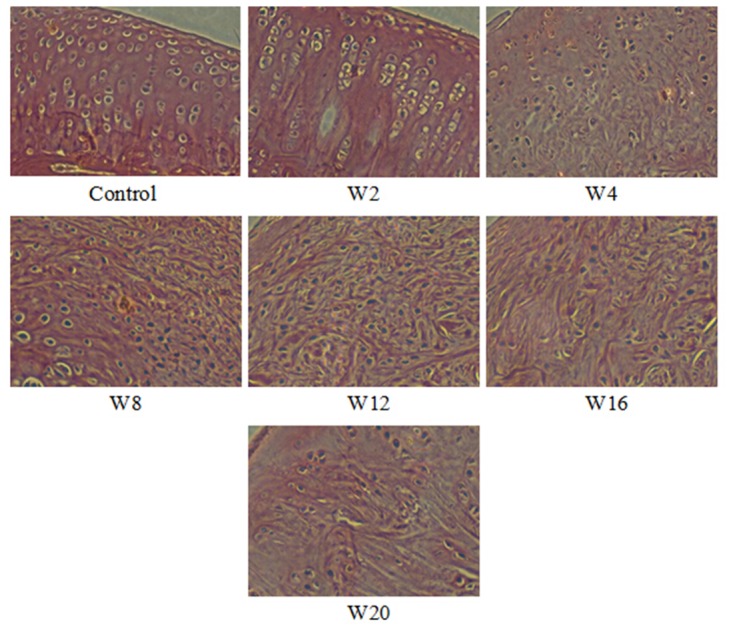
The histological images of the cartilage of control and OA rabbits (HE staining, 20×). Control: Normal cartilage. W2–W20: The cartilage was subjected to the improved Hulth method and evaluated 2, 4, 8, 12, 16, and 20 weeks after surgery, respectively.

After 2 weeks, the group subjected to the improved Hulth method had cartilage tissue with slight injury (Mankin score 1–3). The surface layer was slightly uneven but without cracks. Occasionally, confluent cells were seen. The matrix dye was normal. After 4 weeks, the tissue was slightly injured (Mankin score 3–6). The observations were similar to those of the former group, but the number of confluent cartilage cells increased. The degree of injury became aggravated (Mankin score 7–9) in the 8 week post-operative group, with cartilage fibrosis, cracks involving the interlayer, an irregular tidemark and nonuniform dye of the matrix. After 12 weeks, the injury was worse (Mankin score 8–10) than that observed in the 8 week group. The main characteristics of the cartilage included extension of the fibrosis range, a decrease in the number of cartilage cells, partial disappearance of the tidemark and some loss in the dye of the matrix. After 16 weeks, the cartilage exhibited severe injury (Mankin score 10–12), including the aggravation of cartilage fibrosis, further reduction in the cell number, disappearance of the major tidemark and partial loss in the dye of the matrix. In the last group, the Mankin score range was from 12 to 14. Large-scale fibrosis was observed, and the number of cells decreased greatly. The tidemark disappeared, and the calcified cartilage zone was difficult to distinguish. Most of the matrix dye was lost (Fig 8).

#### The Mankin score results for cartilage histopathology and the correlation with quantitative MRI parameters

**Fig 9:**
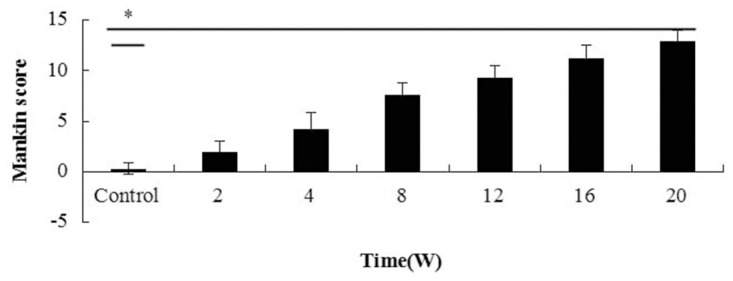
The Mankin score increased at the different time points after surgery with improved Hulth method. Asterisks indicate significant differences.

**Fig 10:**
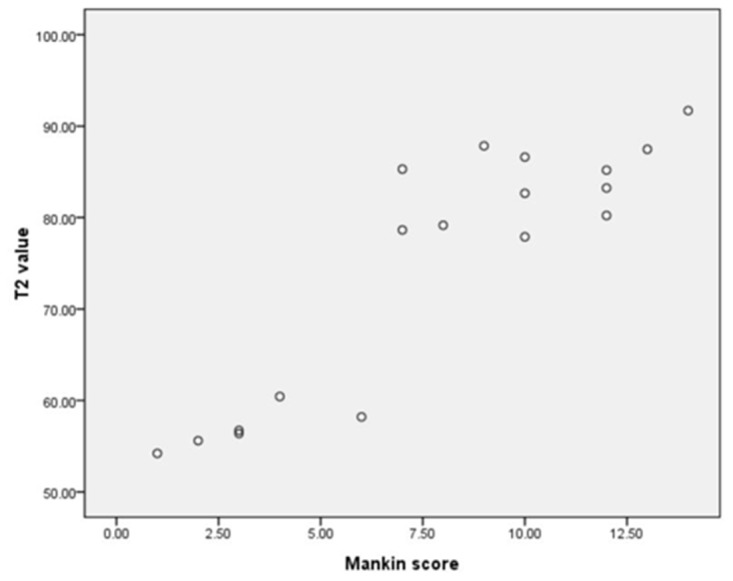
The scatter diagram of T2 values and Mankin scores. A positive correlation was found between the two variables (R^2^ = 0.794, *P*<0.001) using linear regression analysis. A correlation was also found between T2 and T2* values (*P*<0.001). N = 18.

**Fig 11:**
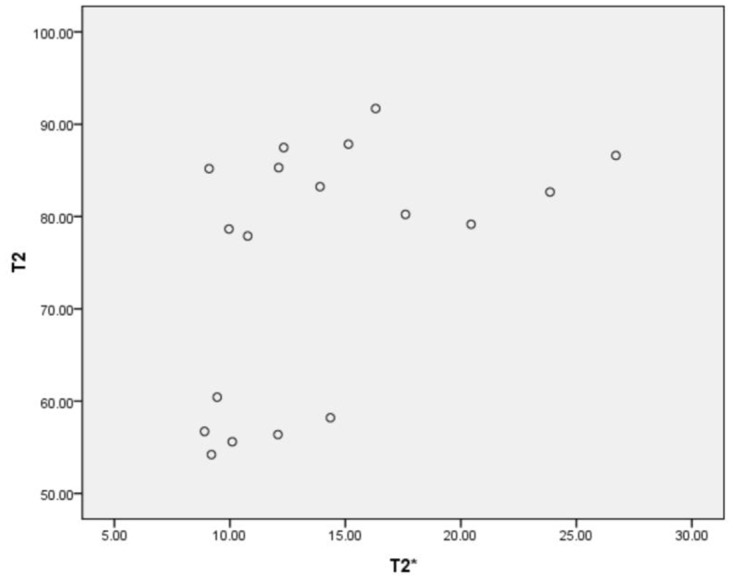
The scatter diagram of T2 values and T2* values. A correlation was found between T2 and T2* values (*P*<0.001). N = 18.

The mean Mankin score of the control was 0.33±0.52. The mean scores of the W2, W4, W8, W12, W16 and W20 OA groups were 2.00±1.00, 4.33±1.53, 7.67±1.15, 9.33±1.15, 11.33±1.16 and 13.00±1.00, respectively. Significant differences were found between the OA groups and the control group (*P*<0.001) (Fig 9). Significant differences were also found between all the OA groups except the 2 and 4 week groups and the 16 and 20 week groups. In the surgically-induced OA rabbits, a positive correlation was found between the T2 values and the Mankin scores (*P*<0.001), while no correlation was found between the T2* values and the Mankin scores(*P* = 0.13) (Fig 10). A correlation between the T2 and T2* values was also found (*P*<0.001)(Fig 11).

#### The correlation between the quantitative MRI parameters and biomarkers

**Fig 12:**
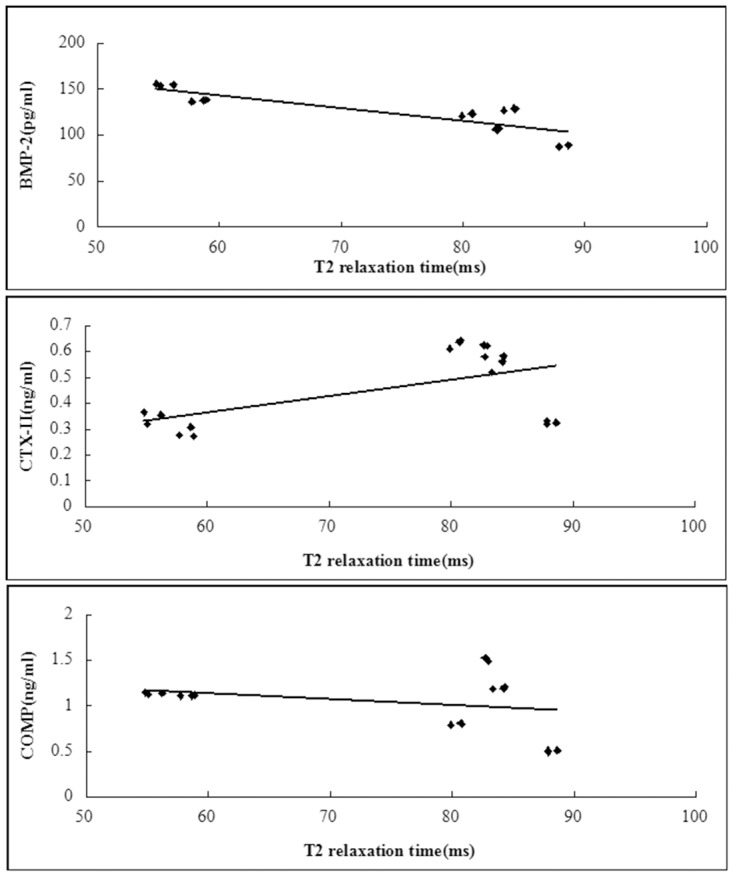
Scattergrams plotting individual serum biomarker concentration against T2 relaxation time values. For T2 relaxation times, there was a linear relationship with BMP-2 and CTX-II, except COMP(A, R^2^ = 0.71, *P*<0.001; B, R^2^ = 0.32, *P* = 0.014; C, R^2^ = 0.07, *P* = 0.305). N = 18.

**Fig 13:**
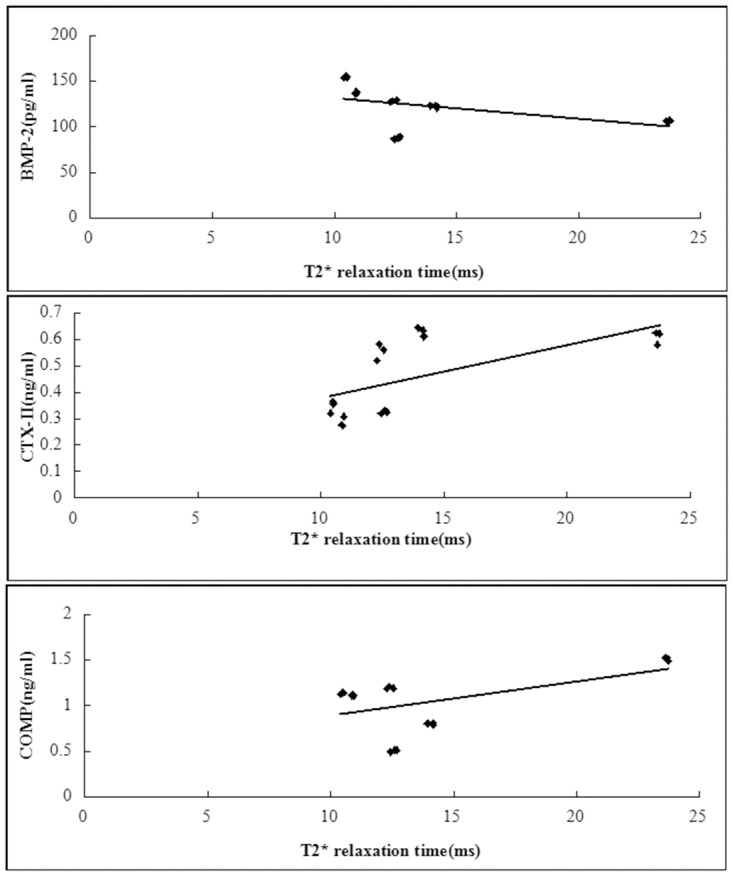
Scattergrams plotting individual serum biomarker concentration against T2* relaxation time values. T2* relaxation times correlated significantly with BMP-2, CTX-II and COMP (D, R^2^ = 0.23, *P* = 0.043; E, R^2^ = 0.4, *P* = 0.005; F, R^2^ = 0.28, *P* = 0.025). N = 18.

The correlations of T2 relaxation times with BMP-2 and CTX-II were significant(*P*<0.001 and = 0.014), except COMP(*P* = 0.305)(Fig 12). The correlation of T2* relaxation times with BMP-2, CTX-II and COMP were significant(*P* = 0.043, 0.005 and 0.025 respectively)(Fig 13).

## Discussion

The goal of this study was to investigate the biomarkers change in serum and the correlation with quantitative MRI markers by histopathologic evaluation of cartilage in the rabbit model of experimental osteoarthritis induced using the improved Hulth method. Our results suggested that these biomarkers played an important role in the process of cartilage repair. The interest of this study was to investigate the correlation between biomarkers and imaging markers. In this study, significant differences of T2 values with BMP-2, CTX-II and T2* values with BMP-2, CTX-II and COMP were observed. To our knowledge, few studies reported the longitudinal evaluation of these biomarkers and the correlation with longitudinal quantitative MRI in an OA rabbit model.

BMP was first extracted from bovine bone by Urist, and the bone formation induction hypothesis proposed in 1965 was theoretically verified [26]. BMP-2 belongs to the TGF-β protein superfamily and has potent osteogenic activity via induction of irreversible mesenchymal cell differentiation into bone and cartilage; BMP-2 also functions in cartilage cell proliferation and repair of injured cartilage [12,27–28]. Fukui et al.[29] reported that BMP-2 is involved in the anabolism pathway in normal and degenerative cartilage, and IL-1β and TNF-α in synovium and cartilage in the setting of osteoarthritis have a synergistic effect with BMP-2. Blaney Davidson et al.[12] found that expression of BMP-2 increased with worsening injury of the cartilage, especially in the later stages. Pfitzner et al.[3] also found that BMP-2 was over-expressed and its concentrations are consequently higher in patients suffering from arthrofibrosis after TKA. We obtained similar results, but the level of BMP-2 was relative lower than the former 3 groups from W12. The concentrations of BMP-2 in the OA groups were higher than in the corresponding control group. The following reasons may account for this phenomenon: i) BMP-2 release in the cartilage was enhanced after injury. ii) The biological behavior of BMP-2 through its involvement with the induction of cartilage cell proliferation and repair of injured cartilage was possibly involved [27–28,30]. In the present study, the concentration of BMP-2 in the 2 week post-operative group was the highest, and the values decreased with the extension of post-operation time, except W16 group. The lowest levels occurred in the 20 week post-operative group. This results suggested that repair of injured cartilage was enhanced in the early stage of cartilage injury. With the progression of cartilage injury, the amount of cartilage tissue and number of cells decreased progressively so that the repair mechanisms could not compensate for it. Therefore, we postulated that the level of BMP-2 could reflect the degree of cartilage injury and the repair progress, especially in the early stage.

C-telopeptide of type II collagen (CTX-II) is produced by type II collagen through the action of proteases and accumulates in urine by circulation as a micro-molecule polypeptide [31–32]. The level of CTX-II may be elevated with cartilage injury or degeneration [33–36]. Therefore, CTX-II may be useful as a biochemical marker for quantifying cartilage degradation in OA and provide a sensitive method to detect increased degradation of collagen type II in patients with osteoarthritis. Duclos et al.[5] investigated articular cartilage degeneration with the detection of serum CTX-II levels in a 5-month longitudinal study in an ACLT surgical model. They found that a first CTX-II peak appeared during the first 3 weeks after surgery. From the sixth week on, a second rise in CTX-II that peaked at W12 for the majority of rabbits was observed, which was followed by a progressive decrease until the end of the study. Therefore, they concluded that aggravated degradation occurred 6 weeks after surgery during the progression of OA disease, and the CTX-II peak might be related to the ulcer lesion due to high cartilage destruction activity. Lohmander et al.[32] found an increase in CTX-II in synovial fluids of patients with knee injury. Other researchers also reported the transient enhancement of CTX-II levels in rabbit OA models [37–38]. Matyas et al.[39] found that the serum CTX-II level was higher in a canine experimental osteoarthritis model than in the pre-surgery group. In our study, the first peak in CTX-II appeared during the first weeks, and the second peak arose between 8 to 16 weeks after surgery, which was slightly different from the previous results. On one hand, the injured cartilage after 2 weeks released more CTX-II, on the other hand, the cartilage volume decreased. These two factors might result in the slow increase in CTX-II at W4. With the severity of OA, the continuous destruction of collagen, more CTX-II was released leading the high level of CTX-II from W8 to W16. So CTX-II could be considered as a potential biomarker for monitoring the progression of OA [5,14].

Cartilage oligomeric matrix protein (COMP) is a tissue-specific protein that is expressed only in cartilage. It may be an index for early OA diagnosis and related to the severity of OA [8, 40–42]. In the development of OA, the COMP levels are obviously enhanced in serum and synovial fluid. El-Arman et al [42] reported that the serum and synovial fluid COMP levels were elevated and positively correlated with radiological joint damage in OA. So, COMP had the potential to be useful for monitoring articular cartilage destruction and the response to different therapeutic modalities. A similar result was found in knee joint OA by Clark et al [43]. In the present study, we obtained results that were similar to those of previous studies. The serum level of COMP was elevated from W2 to W16 post-surgery, and the significant differences were found compared with the control group. After 20 weeks, the COMP level was close to the level of the control group due to the severe destruction and decrease in the volume of the cartilage, the significant difference was still found. From previous and this study, COMP could reflect and monitor the early OA.

In this study, we also delineated the MRI features of OA cartilage and performed T2 and T2* evaluation. The T2 values increased from W8 post-surgery and T2* values increased from W2 to W20. The first peak of T2 values appeared at 8 weeks after surgery, whereas the T2* value peak appeared at 12 weeks. However, the values of T2 and T2* were still relatively higher than those of the control group at W16 and W20. The possible reasons are as follows: i) local magnetic sensitive effect changes due to the great loss of cartilage tissues and the extension of fibrosis in the late stages of cartilage degradation; ii) a partial volume effect that results in the enhancement of values because of exposure of subchondral bone and the replacement of the defect with synovial fluid. In general, the range of the T2* relaxation values was narrower than that of the T2 relaxation values. Mamisch et al. reported that the T2* values were steadily less than the corresponding T2 values, with a mean T2* value that was only 43% of the mean T2 value for healthy tissue. This was reflected in the cartilage repair tissue after microfracture therapy(MFX), where the average T2* value was 41% of the T2 value. They also found that the relative decrease in the relaxation times of repair tissue was greater for T2* (21%) than for T2 (15%)[21].

It was widely studied and reported the T2 and T2* values can reflect the change of cartilage compositions, especially collagen. Many studies used T2 mapping to investigate the biochemical and signal changes in the damaged cartilage and repair tissues after injury. Dardzinski et al. observed a reproducible pattern of elevated T2 values that was proportional to the known spatial variation in cartilage water [44]. Mosher et al. reported increasing T2 relaxation times values in injuried cartilage compared to the healthy [45]. Many studies reported and confirmed T2 mapping can detect the differences in cartilage repair tissue after different repair procedures. In particular, zonal T2 measurements revealed differences between healthy cartilage and cartilage repair tissue in subjects after matrix-associated autologous chondrocyte transplantation (MACT)[46–48]. T2* values and T2 values had similar responsiveness to the properties of articular cartilage and pattern of spatial dependency, but T2* values were consistently less than the corresponding T2 values. According to our findings, T2 values had significant correlation with these biomarkers except COMP, while the correlations of T2* values with these biomarkers were significant. There was some relationship between the serum biomarkers and MR markers, especially CTX-II. Regarding no significant correlation between T2 relaxation time and COMP, while T2* in versus, the following reasons might be involved: i) high specificity and low sensitivity in the use of serum COMP level [49], especially in the later stage of OA. From histopathology, the cartilage dispayed severe injury, including the aggravation of cartilage fibrosis, further reduction in the cell number, disappearance of the major tidemark and partial loss in the dye of the matrix from W16. From W16, the COMP level and T2* values dropped while T2 values was increasing. ii) regional variation in T2* reflects a contribution from several factors, including regional variation in T2 and regional variation in the microstructure of the cartilage. Injury to the calcified cartilage zone and any changes in the collagen architecture of the anisotropic collagen fibers of the deep radial zone could lead to changes in the magnetic susceptibility of the tissue that could be exploited using T2* mapping [21]. iii) T2 and T2* relaxation time measurements have great properties in the evaluation of cartilage repair tissue and its zonal variation, but the properties visualized by T2 and T2* may differ [50].

In our study, we performed the pathological assessment and Mankin score evaluation of the cartilage. In our study, the stage of the OA or the cartilage was based on the classification of Mankin and his colleagues. There are 4 stages based on their Mankin scores: nearly normal (0≤Mankin score<2), early OA (2≤ Mankin score<6), moderate OA (6≤ Mankin score <10), and late OA (10≤Mankin score≤14) [51,52]. In this study, according to the Mankin scores, W2 and W4 was the early stage OA, W8 and W12 was the moderate stage OA, W16 and W20 was the late stage OA. In order to evaluate the OA predictive value of these biomarkers and MRI markers, we performed correlations between them. We also investigated the correlation of T2, T2* values and Mankin scores. A significant correlation between T2 and T2* has been reported [19,53] for the similar responsiveness to the properties of articular cartilage and the similar pattern of spatial dependency, and we obtained the similar results. To our knowledge, few studies reported the correlation between T2 and T2* values and Mankin scores. We found that the correlation of T2 values with Mankin scores was significant. In contrast, no correlation was obtained between T2* values and Mankin scores. This might be due to the changes in the magnetic susceptibility of the tissue caused by regional variation in T2 and regional variation in the microstructure of the cartilage.

There were some limitations or shortcomings in our study. Firstly, high specificity and low sensitivity in the use of serum COMP level in OA diagnosis [49]. Secondly, the T2 and T2* values were susceptible to many factors in the late stages of cartilage degradation, such as volume effects, although some measures were taken to minimize the effect; the sample size was relative small. Thirdly, MR images were not very clear. Many factors had some adverse effect on the results, such as partial volume effect, motion artifact and inhomogeneity of magnetic field and so on. Fourthly, some post-imaging measurements which could have significantly enhanced the level of detail and novelty of this study were not performed, like cartilage volume or defect surface area. Finally, HE staining was used for histopathology preparation and Mankin’s scores rather than safranin O staining, which was another shortcoming of our study. Improvement of the method, post-imaging measurements, histopathologic evaluation and large sample size are the focus of our ongoing works. In the next step, the therapeutic effect will be investigated with the OA animal models.

In conclusion, we were able to establish experimental osteoarthritis model with the improved Hulth method in this study. Our results indicated that these biomarkers could potentially be used to reflect and monitor OA progression and the relevance of the multiple time point analysis of the biomarkers and imaging markers. T2 and T2* markers in combination with biomarkers showed great promise in the evaluation of cartilage repair tissue and its zonal variation, although the applications of T2 and T2* relaxation time may differ. Our findings may provide useful information for the clinical management and follow-up of OA and lay preliminary foundation of noninvasive observation of OA in the future.
